# Development of Biological Oxygen Demand Biosensor for Monitoring the Fermentation Industry Effluent

**DOI:** 10.5402/2013/236062

**Published:** 2012-11-27

**Authors:** Neelam Verma, Ashish Kumar Singh

**Affiliations:** Department of Biotechnology, Punjabi University Patiala, Punjab, Patiala 147 002, India

## Abstract

A biosensor was developed for the determination of BOD value of fermentation industry effluent. The developed biosensor was fabricated by immobilizing the microbial consortium on cellulose acetate (CA) membrane in close proximity to a DO probe electrode. The microbial consortium was harvested from the fermentation industry effluent. The BOD biosensor was calibrated by using a solution containing the equivalent amount of glucose/glutamic acid (GGA) as a standard sample solution. The response time was optimized by immobilizing different concentrations of cell biomass on CA membrane. Once the response time was optimized, it was used for determination of BOD of fermentation industry effluent. For analysis of fermentation industry effluent, the response time was observed 7 minutes with detection limit 1 mg/L. Good linear range with GGA standard solution was observed, *R* ^2^ 0.99 with relative standard deviation (RSD) <%. The observed BOD value by biosensor showed a good comparison with the conventional method for the determination of BOD.

## 1. Introduction

Biochemical oxygen demand (BOD) is one of the most important and widely used parameters for characterizing the organic pollution of water and wastewater, which is estimated by determining the amount of oxygen required by aerobic microorganisms for degrading organic matters in wastewater. Conventional BOD method is the well-known BOD_5_ which needs 5-day incubation at 20°C in the dark [1].

The United States includes BOD effluent limitations in its secondary treatment regulations. Secondary sewage treatment is generally expected to remove 85 percent of the BOD measured in sewage and produce effluent BOD concentrations with a 30-day average of less than 30 mg/L and a 7-day average of less than 45 mg/L. The regulations also describe “treatment equivalent to secondary treatment” as removing 65 percent of the BOD and producing effluent BOD concentrations with a 30-day average less than 45 mg/L and a 7-day average less than 65 mg/L [1]. Most pristine rivers will have a five day carbonaceous BOD below 1 mg/L. Moderately polluted rivers may have a BOD value in the range of 2 to 8 mg/L. Municipal sewage that is efficiently treated by a three-stage process would have a value of about 20 mg/L or less. Untreated sewage varies but averages around 600 mg/L in Europe and as low as 200 mg/L in the US, or where there is severe groundwater or surface water infiltration. The generally lower values in the US derive from the much greater water use per capita than in other parts of the world [2]. 

### 1.1. Calculation of BOD Value

The BOD of a waste water can be defined as the amount of oxygen expressed in milligrams per liter required by the microorganism for the biodegradations of the degradable carbonaceous organic matter present in the water through their biochemical, bioprocess, and under the following reaction conditions: temperature 20°C, five-day retention time, and darkness to avoid the presence of microscopic algae that produce oxygen by photosynthesis thus interfering with the result. Because the saturation conc. for oxygen in water at 20°C is approximately 9 mg/L dilution of the sample with BOD free, oxygen-saturated water is necessary to measure BOD values greater than just a few mg/L. BOD of a diluted sample is calculated as

(1) $$\begin{array}{c} \text{BOD}=\frac{{\text{DO}}_{\text{I}}-{\text{DO}}_{\text{F}}}{P}, \end{array}$$

where DO_I_ and DO_F_ are initial and final dissolved oxygen concentrations (mg/L) and *P* is the decimal fraction of the sample in 300 mL bottle [3].

Biochemical oxygen demand (BOD) is an important index for monitoring organic pollutants in water. The conventional standard method (five day BOD test, BOD_5_), however, is a complicated and a time-consuming procedure, including a five-day incubation, and also requires considerable experience and skill to get reproducible results [4]. Fast determination of BOD could be achieved by biosensor-based methods. A common feature of these sensors is that they consist of a microbial film that can biooxidize the organic substrate to be quantified, sandwiched between a porous cellulose membrane and a gas-permeable membrane as the biological recognition element. First BOD biosensor was developed by immobilization of *Trichosporon cutaneum* on the oxygen electrode [5]. Some BOD sensors have been developed and marketed by various manufacturers in both, biofilm and bioreactor-type configurations. Most commercially available BOD sensors are flow-type systems that can be more easily automated but generally require high maintenance to prevent fouling and clogging [6]. The response is usually a change in concentration of dissolved oxygen or other phenomena such as light emission [7].

Despite the good agreement between biosensor results and conventional BOD analysis, and despite the short response time of biosensors, current BOD biosensor systems still present a series of limitations that restrict their industrial applications: the lack of standardization and legislation in most countries, complicated maintenance requirements, and insufficient resistance to various toxic compounds such as heavy-metal ions, CN^−^ ions, and phenol in the wastewater. It is possible to eliminate the toxic effects of heavy-metal ions by using a chelating agent that complex the ions, for example, ethylene diamine tetra-acetate (EDTA) and sodium diethyl dithiocarbamate [8, 9]. Prevention of contamination by other microbes is also important for a reliable biofilm-type BOD sensor [7].

Literature survey reveals many biosensors developed for BOD determination by using the different type of biological components and also different strategies of immobilization techniques. Qian and Tan [10] used heat-killed *B. subtilis* for BOD determination and stability of their operation is about 140 days. The immobilized *Pseudomonas putida* bacterium membrane was placed on the top of an optode, which was linked to a photo diode that detected fluorescence signal. The response time was 15 min for chloride up to 1000 mg/L [11]. Many BOD biosensors have been developed for the determination of high BOD values in industrial wastewater and not adapted to the measurement of low BOD values. An optical fiber biosensor was developed for the evaluation of low BOD values in river waters [12]. BOD sensor system of flow injection mode, constructed by combining an immobilized microbial reactor with an electrochemical flow cell of three electrodes configuration, has been developed to estimate BOD [13]. BOD sensor based on immobilizing multispecies BOD seed for wastewater monitoring has been developed in the flow system [14]. A novel reactor-type biosensor for rapid measurement of BOD was developed, based on using immobilized microbial cell (IMC) beads as a recognition bioelement in a completely mixed reactor [15]. Some online system for determining organic pollutants by using biofilm-reactor-based approach [16]. A BOD biosensor based on the microbial fuel cell principle was tested for online and in situ monitoring of biodegradable organic content of domestic wastewater [17]. Some other kind of biosensor was developed for the amperometric short time BOD analysis by applying microfluidic respirometer [18]. A biosensor was developed for the determination of BOD value of specific industry effluent such as for meat industry effluents [19], paper and pulp industry effluents [20]. Instead of bacterial cell some yeast cells were also used for the construction of BOD biosensor [21].

In this study, we propose a new analytical approach that has very cost-effective and simple idea for determination of BOD value of fermentation industries effluents. This new approach utilizes microbial consortium that was isolated from fermentation industry effluent and separately immobilized on the cellulose acetate membrane. The primary objective was isolation of microbial consortium, immobilization on transducer, that is, DO probe, optimization of response time, and comparing BOD by using this biosensor with Winkler's method (BOD_5_).

## 2. Materials and Methods

### 2.1. Chemical Reagents

Phosphate buffer was prepared by dissolving (KH_2_PO_4_ 8.5 g, K_2_HPO_4_ 21.75 g, Na_2_HPO_4_ 33.4 g, NH_4_Cl 1.7 g) in 1 L water, ferric chloride (FeCl_3_·6H_2_0) 0.25 g/L, calcium chloride (CaCl_2_) 27.5 g/L, magnesium sulphate (MgSO_4_·7H_2_O) 22.5 g/L dilution water was prepared by adding 2 mL of each of the above reagent. Some other chemical reagents as manganese sulphate solution (MnSO_4_·4H_2_O), alkaline-iodine reagent (dissolved 500 gm of NaOH and 135 gm of NaI or 700 gm KOH and 150 gm KI), were added in distilled water and diluted to 1 liter. Add 10 gm of NaN_3_ dissolved in 40 mL of distilled water), concentrated H_2_SO_4_, starch (1 gm of starch was dissolved in 100 mL distilled water), standard sodium thiosulphate (0.025 N dissolved exact weight of 9.205 gm anhydrous sodium thiosulphate in distilled water and diluted it to 1 liter), and Nutrient Broth M002 (HiMedia, Mumbai). All chemicals were purchased from Sigma (St. Louis, USA) and used in analytical or higher grade. Cellulose acetate membrane (0.2 OE-66) was purchased from Whatman part of GE Healthcare, India.

### 2.2. Apparatus

DO sensor amperometric gold/silver membrane type (DO range 0 to 40.0 ppm and temperature range 0 to 500°C) and its Resolution accuracy (DO 0.1 ppm, Temperature 0.1°C), and temperature sensor RTD (PT-100) make of Labtronics Inc., Ontario, Canada.

### 2.3. Procurement of Industrial Effluent and Its Characteristics

Fermentative industry effluent was procured from Patiala distiller and manufacturers situated at Village-Main, District-Patiala, Punjab (India), and characteristic features of industrial effluents are given in Table 1.

### 2.4. Isolation of Microbial Consortium

The microbial consortium isolated in nutrient broth medium (readymade) by using 2% inoculums (effluent from fermentative industry) was used. After inoculation the culture medium was kept on shaker incubator at 37°C for 24 hours. Subculturing was done again in nutrient broth at 37°C for 24 hours, and culture was maintained in the same medium every week and 2% of inoculum was used. 

### 2.5. BOD Determination by Conventional (Five-Day) Method

The five-day BOD of sample was determined by Winkler's method [22].

Calculations:

(2) $$\begin{array}{c} \text{DO}\left(\text{mg}/\text{L}\right)\;=\frac{V\times N\left(\text{Hypo}\right)\times 8\times 1000}{\text{Volume}\;\text{of}\;\text{sample}} \end{array}$$

*V* = volume of Na_2_S_2_O_3_ used, *N* = normality of Na_2_S_2_O_3_ (Hypo) (0.025N), 8 = equivalent weight of oxygen. The test sample was prepared by diluting the I.F. and in water has free of oxygen and microbes, and the final test sample 10% of I.F. All experiments were done in triplicate, and their average values are considered.

### 2.6. Operation and Calibration of DO Probe

Oxygen dissolves in water, often referred to as DO; the main source of DO in water is diffusion from air and photosynthetic activity. Nonpolluted surface water is normally saturated with DO. First, temperature was set according to solution temperature by “temperature knob.” Place the DO probe in 2% sodium sulphite solution (solution having no oxygen because it's absorbed by sodium sulphite), allow the display to attain equilibrium, and then set the zero by “zero knob.” It calibrated the instrument with known value of DO solution (fully agitated with oxygen-distilled water at different temperature) that was used (Table 2).

Hold DO probe in flask containing saturated distilled water and agitate the water and if necessary adjust the meter reading with “cal” knob. Now instrument was ready to determine the DO of unknown solution.

### 2.7. Immobilization of Microbial Consortium on CA Membrane

The basis of a microbial biosensor is the close contact between microorganisms and the transducer. Thus, fabrication of a microbial biosensor requires immobilization on the transducer with a close proximity. Since microbial biosensor response, operation stability, and long-term use are, to some extent, a function of the immobilization strategy, the immobilization technique is the physical method in which membrane entrapment of whole cell was achieved by using cellulose acetate membrane. First, the cells were harvested from a culture medium by centrifuge at 6000 RPM, then suspension was made in the phosphate buffer pH 7.4 that showed OD 1.0 at 600 nm. At this stage the cell concentration was found to be 1 × 10^8 ^ cfu/mL. Further this cell biomass was used for immobilization on cellulose acetate membrane at different cell concentration.

### 2.8. Calibration of BOD Biosensor and Response Time Optimization

BOD biosensor was calibrated using standard solution having the equivalent amount of glucose and glutamic acid (GGA) 150 mg/L, with BOD value of 220 mg/L ± 11.0. Optimization of response time, that is, 0, 4, 8, 12, 16, 20, 24, 28, and 30 minutes for microbial degradation of the equivalent amount of GGA solution by the microbial consortium was studied. After response time optimization, the linear range of BOD biosensor was also studied by using different concentration range (1–150 mg/L) of GGA standard solution.

### 2.9. BOD Determination by Biosensor and Comparison with BOD_5_

100 mL diluted sample was taken, an initial DO was found by dipping the oxygen electrode and after that oxygen, electrode was coupled with cellulose acetate membrane containing immobilized microbial consortia and found the final DO after a particular time (response time), and calculated the BOD value of respected sample (fermentation industry effluent). The BOD value observed by biosensor was also compared against the BOD value determined by the conventional method of same sample.

## 3. Results and Discussion

### 3.1. Estimation of BOD Value of Effluent Sample

The five-day BOD of sample was determined by Winkler's method. The value of BOD of fermentative I.F. determined by conventional method was found 180 ± 10 mg/L (Table 3).

### 3.2. Optimization of Response Time

The response time (time taken by microbial cell to oxidize the GGA solution) was studied by using different volumes of cells (microbial consortia) biomass at absorbance = 1, that were immobilized on cellulose acetate membrane. Each immobilized membrane was coupled with the probe and tested against the GGA standard solution, and decrease in DO was observed (Table 4).

**Figure 1:**
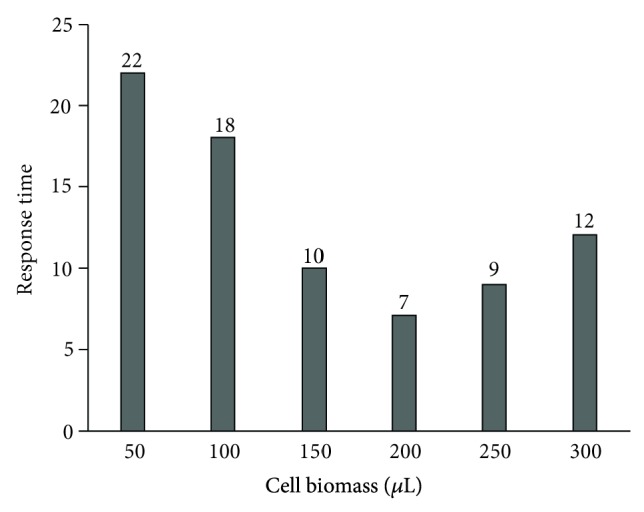
Comparison of response time with cell biomass of microbial consortium.

The response time with cell biomass of microbial consortium concentration 2 × 10^7^ cfu/mL was observed only 7 minutes (Figure 1).

**Figure 2:**
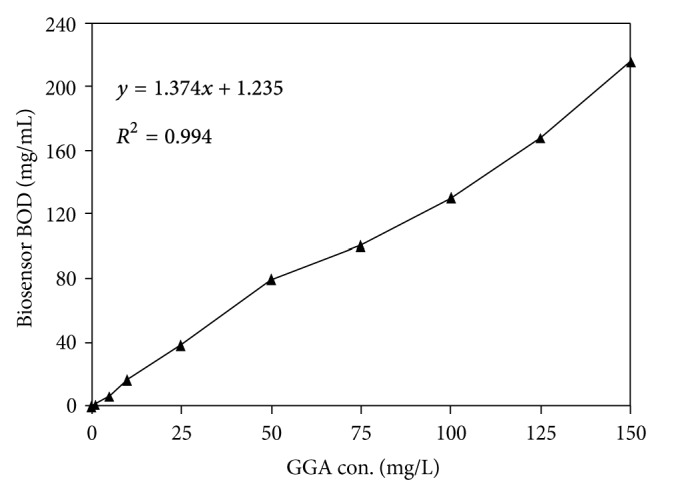
Linear range of biosensor BOD of standard solution of GGA at different concentration (*N* = 5) with RSD < 9%.

K*ӧ*nig et al. [23] developed BOD biosensor for determination of BOD for nitrification (N-BOD) by using nitrifying bacteria immobilized at an oxygen electrode, and response time was about 12 minutes. Microbial fuel cell (MFC) was used in the determination of BOD value of waste water; the minimum detection limit of this MFC is about 0.2 mg/L with a response time of two hours [24]. Chen et al. [25] developed BOD biosensor that was also developed with response time of 15 minutes and also found linear relationship between the response (sensor current) and BOD values ranging from 10–15 mg/L. At higher cell concentrations, the response time was again increased, because the layer of immobilized cell on membrane is thick, and they cause a low rate of consumption of organic substances. Cheng et al. [26] used luminescent bacteria *Vibrio fischeri, Photobacterium phosphoreum,* and recombinant *Escherichia coli* as potential indicators of BOD in the domestic waste water and response time for biosensor 90, 120, and 150 minutes, respectively. In the present study, the linear range of developed BOD biosensor was also observed with different concentration of standard GGA solution. We found a good correlation coefficient (*R* ^2^) about 0.99 with RSD < 9%, and also the detection limits were found 1 mg/L (Figure 2).

### 3.3. Comparison of BOD_5_ and BOD Estimated by Biosensor

Biosensors constructed by using microbial consortium were tested for determination of BOD value of fermentative I.E. As shown in Table 5 relatively good agreement between the two methods was obtained for the test sample with relative error ± (6–8). BOD biosensor has also shown good agreement between the results of the sensor BOD measurement and those obtained from conventional BOD_5_ analysis [27]. The experiments were done in triplicate, and the average values are given.

The developed BOD biosensor is reliable, economical because here we use the microbial consortium that was isolated from the fermentation I.F., and one advantage of this biosensor is their wide range of application for different types of waste water because the microbial consortia are capable to degrade an extensive range of organic pollutants present in waste water. Earlier developed BOD biosensor based on specific microorganism has major disadvantage regarding their narrow range of degradation of organic compound [14].

## 4. Conclusion

In this study, a microbial electrode biosensor consisting of immobilized living whole cells on cellulose acetate membrane and oxygen probe has been developed for the estimation of biochemical oxygen demand (BOD). Immobilized microbial consortium isolated from the fermentation industry effluents was employed for the microbial electrode sensor for BOD. This BOD biosensor was calibrated by using a solution containing the equivalent amount of GGA (BOD = 220 mg/L) as a standard sample solution and optimized the response time. Once response time optimized, it was used for determination of BOD of fermentation industry effluent sample. The response time of microbial consortium was very fast only 7 minutes to give BOD value of fermentation industrial effluent. BOD value of fermentation industry effluent was also determined by conventional 5-day methods. The comparison of both BOD value, one BOD value from BOD biosensor and other from the conventional 5-day methods, shows good comparable results.

## Figures and Tables

**Table 1:** Characteristic features of industrial effluents.

Parameter	Untreated effluent	Treated effluent
	(mg/L)	(mg/L)
BOD	3000–3500	120–180
COD	6200–7600	300–340
TSS	70–75	45–50

Total “*N*”	80–90	30–35

**Table 2:** DO level of saturated distilled water at different temperature.

Temperature (°C)	DO (ppm)
0	14.32
5	12.8
10	11.6
15	10.9
20	9.00
22	8.67
24	8.36
26	8.06
28	7.76
30	7.50
32	7.30
35	7.00

**Table 3:** Estimation of BOD value of fermentative industrial effluent by conventional method.

Effluent	Initial DO	Final DO	BOD_5_
(fermentative I.E.)	(mg/L)	(mg/L)	(mg/L)
10%	4.6	1.8	180 ± 10

*N* = 3.

**Table 4:** Showing effect of cell biomass on its response time.

Cell biomass (cfu/mL)	Response time (min)
0.5 × 10^7^	22
1.0 × 10^7^	18
1.5 × 10^7^	10
2.0 × 10^7^	7
2.5 × 10^7^	9
3.0 × 10^7^	12

**Table 5:** Comparison between conventional method and BOD biosensor for fermentative industrial effluent bacterial isolate.

Fermentative I.E.	BOD (mg/L)	
	BOD_5_	BOD biosensor
5%	180 ± 12	170 ± 8
10%	180 ± 10	190 ± 6

*N* = 3.
